# Cognitive Spare Capacity and Speech Communication: A Narrative Overview

**DOI:** 10.1155/2014/869726

**Published:** 2014-05-27

**Authors:** Mary Rudner, Thomas Lunner

**Affiliations:** ^1^Linnaeus Centre HEAD, Swedish Institute for Disability Research, Department of Behavioural Sciences and Learning, Linköping University, 581 83 Linköping, Sweden; ^2^Department of Clinical and Experimental Medicine, Linköping University, 581 83 Linköping, Sweden; ^3^Eriksholm Research Centre, Oticon A/S, 3070 Snekkersten, Denmark

## Abstract

Background noise can make speech communication tiring and cognitively taxing, especially for individuals with hearing impairment. It is now well established that better working memory capacity is associated with better ability to understand speech under adverse conditions as well as better ability to benefit from the advanced signal processing in modern hearing aids. Recent work has shown that although such processing cannot overcome hearing handicap, it can increase cognitive spare capacity, that is, the ability to engage in higher level processing of speech. This paper surveys recent work on cognitive spare capacity and suggests new avenues of investigation.

## 1. Introduction

Speech is the main mode of communication for most people. If speech understanding is compromised by noise or hearing impairment, communication may become harder, leading to limitations in social participation. Technical compensation is available in the form of hearing aids. However, although the amplification provided by hearing aids can improve speech understanding in quiet, persons with hearing impairment still have disproportionately large difficulties understanding speech in noise. One of the reasons for this may be that when the cognitive resources required for speech comprehension are engaged in the lower level processes of deciphering the signal, fewer resources may be available for higher level language processing. In other words, cognitive spare capacity is reduced.

### 1.1. Speech Comprehension

Speech comprehension requires the auditory ability to hear the signal and the cognitive ability to relate this information to the existing knowledge stored in semantic long-term memory [1, 2]. The role of cognition in speech comprehension is reflected in the hierarchical nature of its cortical representation [3, 4].

Speech processing engages a clearly defined cortical network involving the classical language areas in the left inferior frontal cortex and superior temporal gyrus [3, 4]. The primary auditory cortex is sensitive to most sounds and is the first cortical region to be activated during speech perception [4]. Listening to words activates the middle and superior temporal gyri bilaterally and listening to sentences engages regions involved in processing semantics and syntax in the left prefrontal cortex [3]. It has been possible to trace the pathways linking these regions by using animal models [4–6]. These pathways represent different functional streams that take either a ventral route through superior temporal regions to ventrolateral prefrontal cortex or a dorsal route through posterior parietal cortex and dorsolateral prefrontal cortex [6, 7]. One ventral route seems to deal more with conceptual or semantic processing, while there is a dorsal route that is more related to phonological or articulatory processing [6, 7]. Ventral and dorsal routes for syntactic processing have also been proposed [8].

### 1.2. Hearing Impairment

Around 25% of the population in developed countries has a hearing impairment severe enough to interfere with speech communication [9]. Hearing sensitivity decreases with age such that although only about 2% of individuals in their early twenties have a hearing loss, the prevalence of significant hearing impairment is 40–45% in persons over the age of 65 and exceeds 83% in persons over the age of 70 [10, 11]. Hearing difficulties are associated with long-term absence from work in the working age population [12, 13] and loneliness in the older population [14]. Further, individuals with better cognitive abilities report more hearing difficulties [15, 16], possibly because they have higher expectations of their communication. Even moderate degrees of hearing impairment lead to decrease in neural activity during speech processing and may contribute to grey matter loss in primary auditory cortex [17, 18].

Types of hearing loss are traditionally categorized according to site of lesion: impairment of sound transmission in the external or middle ear is referred to as conductive hearing loss, while other types of hearing loss are referred to as sensorineural. Sensorineural hearing loss can be further subdivided into sensory loss, resulting from impairment of cochlear function, retrocochlear loss, resulting from impairments relating to conduction in the auditory nerve or brainstem, and central losses, resulting from impairments in cortical processing of the auditory signal. Sensorineural hearing loss is the major diagnostic category and includes age-related hearing loss or presbyacusis. These categories are relatively coarse and it has been suggested that they may be inadequate for pinpointing the contribution of hearing loss to communication difficulties under adverse listening conditions [19].

The primary diagnostic tool in audiology is the pure tone audiogram. This method of determining frequency-specific hearing thresholds is based on delivering sine waves of different intensities to each ear and asking the patients to respond by pressing a button each time they hear a sound. The resulting resolution is poor, and since this procedure requires the processes of intention and attention that characterize listening as opposed to simply hearing and thus tap into cognitive processes that may also be declining with age, diagnosis may be confounded. Other diagnostic tools include measures of auditory brainstem response and otoacoustic emissions which may be more independent of high-level cognitive contribution, although it has recently been shown that cognitive load influences brainstem responses [20] and otoacoustic emissions may also be influenced by attention through efferent innervation [21]. Assessment of speech intelligibility in quiet and in noise is also part of hearing evaluation.

### 1.3. Hearing Aids

The most important objective for hearing aid signal processing is to make speech audible [22]. This is not a trivial problem. Over 30 years ago, Plomp [23] proposed a model of hearing aid benefit that classed hearing impairment in terms of attenuation and distortion showing that while the hearing aids of the day could compensate well for the former by providing amplification, they were poorer at tackling the latter. As distortion is a characteristic of even the mildest hearing losses, it is important that hearing aids address this issue and the industry has taken on this challenge [24]. Distortion can be simply characterized as a decrease in the ability to distinguish speech from noise. It is not only due to decreased frequency and temporal resolution, as well as impaired ability to discriminate pitch and localize sound sources, but also due to abnormal growth of loudness [25], such that if all sounds are amplified the same way, some may become uncomfortably loud. Thus, modern digital hearing aids include technologies that tackle some of these problems [26]. Wide dynamic range compression systems restore audibility by amplifying weaker sounds more than loud sounds to compensate for the abnormal growth of loudness. The regulation of the compression system may be fast (syllabic) or slow (automatic volume control). Fast-acting wide dynamic range compression (fast WDRC) provides different gain-frequency responses for adjacent speech sounds with different short-term spectra on a syllabic level. On the assumption that communication partners look at each other, directional microphones may be used to attenuate sounds not coming from the front. Of course, if the attended signal does not come from the front, directional microphones may make communication harder. Single-channel noise reduction schemes (NR) may reduce background sounds by identifying portions of the signal as nonspeech and attenuating these. This does not improve speech intelligibility per se, but it may reduce the annoyance from background sounds. Notwithstanding the benefits of signal processing, there is no getting away from the fact that it may also degrade the auditory signal, which may make listening harder. This applies in particular to aggressive signal processing algorithms that may be used experimentally but are not generally prescribed to patients. Aggressive processing is characterized by substantial spectral alteration of the signal within the space of a few milliseconds. For example, some aggressive NR algorithms generate audible artifacts [27] and WDRC distorts individual speech sounds in ways that influence the phonological or sublexical structure of the incoming speech signal [22, 28–30].

### 1.4. Noise

Acoustic noise impacting speech perception can be categorized as signal degradation, energetic masking, and informational masking [31]. Signal degradation reduces the amount of information in the signal. As we have seen, this is the result of hearing aid signal processing. Other examples relate to processing for data transmission. Energetic masking is a competing signal that partially obscures the target signal. Air conditioning fans are a good example. Informational masking also obscures the target signal but in addition has a fluctuating structure that in some circumstances may distract the listener but in others may allow the listener to systematically glimpse parts of the signal. An informational masker may consist of tonal patterns, for example, or one or more competing speakers. As regards the neural networks underpinning speech comprehension in noise, a pattern is starting to emerge involving widespread frontal and parietal activation as well as increased temporal activation [32]. There is also some evidence that the brain tracks target and competing speech streams in a manner that is modulated by attention [33] with selective attention networks for pitch and location [34].

Persons with hearing impairment have particular difficulties listening in noise which may be reflected in recruitment of neural networks supporting compensatory processing [35, 36] whereas persons with normal hearing are generally better at coping with informational than energetic masking [37]; the same may not always be true for persons with hearing impairment [38–40]. An informational masker includes cues in terms of pitch or temporal fine structure that may help segregation and dips in the masker may reveal portions of the target signal. This may result in the listener perceiving fragments of a target signal that need to be pieced together to achieve understanding. An informational masker may also include semantic information that distracts the listener from the target signal and thus needs to be inhibited. Such processes rely on cognitive functions.

## 2. Working Memory and Speech Comprehension

### 2.1. The Role of Cognition in Listening

Cognitive processes are required to focus on the speech signal and match its contents to stored knowledge [1, 2]. When listening takes place in adverse conditions, for example, when there is background noise or the listener has a hearing impairment, high-level cognitive functions such as working memory and executive processes are implicated [41, 42]. Working memory (WM) is the capacity to perform task-relevant processing of information kept in mind [42, 43] and is supported by a frontoparietal network [44, 45] that is sensitive to stimulus quality and memory load [46, 47]. Many different models of WM have been proposed [48], and one of the most influential of them is the component model originating in the seminal 1974 paper by Baddeley and Hitch [43]. This model was characterized by a central executive controlling two slave buffers for processing verbal and visuospatial information, respectively. It elegantly accounted for a host of empirical data from dual task paradigms, that is, tasks requiring two different kinds of processing at the same time. However, it could not easily account for evidence of multimodal information binding, for example, use of visual cues during speech understanding. A new generation of WM models including an episodic buffer filling just such a function saw the light of day around the turn of the 21st century. These include an updated version of the original component model [49] and a model specifically describing the role of WM in language understanding: the WM model for ease of language understanding (ELU) [41, 50]. Although early work placed the episodic buffer among executive functions organized in the frontal lobes [51], later work has shown that multimodal information binding does not necessarily load on executive functions. For example, visual binding has been shown to take place without executive involvement [52] and multimodal semantic binding has been shown to have its locus in the temporal lobes [53, 54].

The ELU model [41] links in with a parallel line of conceptual development represented by the individual differences approach to WM. This approach focuses on the large variance in individual ability to perform WM tasks rather than characterizing different components of WM [55–57]. According to the ELU model [41], language understanding proceeds rapidly and smoothly under optimal listening conditions, facilitated by an episodic buffer which matches phonological information in the incoming speech stream with the existing representations stored in long-term memory. Because this buffer deals with the rapid, automatic multimodal binding of phonology, it is known by the acronym RAMBPHO. Adverse listening conditions hinder RAMBPHO processing. This may result in a mismatch between auditory signal and information in the mental lexicon in long-term memory. Under such circumstances, explicit or conscious processing resources need to be brought into play to unlock the lexicon. The ELU model proposes that this occurs in a slow processing loop. Processing in the slow loop may include executive functions such as shifting, updating, and inhibition [58]. Inhibition may be required to suppress irrelevant interpretations, while updating may bring new information into the buffer at the expense of discarding older information. Shifting may come into play to realign expectations [30, 59]. All these functions are linked to the frontal lobes [44] and there is evidence that they are supported by anatomically distinct substrates [60]. Their role in speech communication under adverse conditions may be bringing together ambiguous signal fragments with relevant contextual information. There is a constant interplay between predictive kinds of priming of what is to come in a dialogue and postdictive reconstructions of what was missed through mismatches with the lexicon in semantic long-term memory [41]. There is no doubt that such processing is effortful and increases cognitive load [61, 62] and modulates the neural networks involved in speech processing under adverse conditions [63]. From an individual difference perspective, it makes sense that individuals with high WM capacity would perform better on tasks requiring speech understanding under adverse conditions, and this is indeed the case [64–66].

More than a decade ago, it was established that there is a relation between cognitive ability, in particular WM capacity, and the benefit obtained from hearing aid signal processing [64, 67–69]. In particular, it was shown that any benefit of fast-acting WDRC in terms of the ability to understand speech in noise was contingent on cognitive ability [64, 68]. Since then, it has been shown that this relationship is influenced by type of background noise [70–72] and the type of target speech material [30, 70, 73]. Cognitive resources are especially important when modulated noise is combined with fast-acting WDRC [30, 61, 71–73] above all when the target speech is unpredictable [30]. These complex relations change over time [30, 73, 74].

The capacity of WM can be increased by training, suggesting an inherent plasticity in the system [75, 76]. Training effects may generalise to similar nontrained tasks, for example, a different WM task [75]. This is known as near transfer. However, generalization to other cognitive abilities, known as far transfer, has been elusive [77]. Recent work, however, has shown that for older adults, cognitive training requiring multitasking can result in sustained reduction in multitasking costs and improvement in WM [78]. As we have noted, WM is about simultaneous storage and processing, in other words a form of multitasking. The results of Anguera et al. [78] suggest that in order to improve WM, it may be more efficient to target multitasking abilities as such. Since WM capacity is related to the ability to understand speech in noise, it is tempting to speculate that increasing WM capacity may also improve the ability to understand speech in noise. However, published evidence for the efficacy of individual computer-based auditory training for adults with hearing loss is not robust [79]. We suggest that cognitive training that targets the multitasking abilities inherent in speech understanding under adverse conditions may improve WM capacity and result in better speech understanding in adverse conditions. This is an important avenue for future research.

## 3. Cognitive Spare Capacity for Communication

### 3.1. Cognitive Spare Capacity

When listening takes place in adverse conditions, it is clear that the cognitive resources available for higher level processing of speech will be reduced [80]. In other words, the listener has less cognitive spare capacity (CSC) [59, 69, 81, 82].

CSC is closely related to WM in that it is concerned with short-term maintenance and processing of information [59]. Work to date suggests that the storage functions of CSC and WM are similar [83] but that once executive processing demands are introduced, there no longer seems to be a simple relationship between the two concepts [69, 82, 84]. Thus, in order to understand the role of cognition in speech understanding under adverse conditions, it is important to measure not only WM capacity but also CSC. The concept of CSC is related to, although distinct from, other concepts in the literature. For example, differences in susceptibility to functional impairment as a result of brain damage have been explained in terms of “cognitive reserve,” that is, individual differences in cognitive function [85], or “brain reserve,” that is, individual differences in brain size [86]. CSC is similar to these concepts in that it is based on individual differences in cognitive function and may explain differences in speech communication and underlying mechanisms that may be related to functional changes at any level of the auditory system [69, 81].

Recent work has shown that noise reduction (NR) in hearing aids can enhance CSC by improving retention of heard speech [83, 87]. This applies to both adults with normal hearing thresholds [87] and adults with sensorineural hearing impairment [83]. In the study by Ng et al. [83], experienced hearing aid users listened to sets of highly intelligible, ecologically valid sentences from the Swedish hearing in noise test (HINT) [88, 89]. The HINT sentences were presented in noise and the participants were asked to memorize the final word of each sentence. The participants repeated all the target words to ensure that they were intelligible. At the end of each set, participants were prompted to recall all the sentence-final words. Although they were capable of repeating the sentence-final words, irrespective of the presence of background noise, noise did disrupt recall performance [83]. Being able to retain heard information is an integral part of speech communication. Thus, the findings of Ng et al. [83] demonstrate that, for individuals with hearing impairment, background noise reduces the cognitive resources available for performing the kind of cognitive processing involved in communication. This is in line with the work showing that extra effort expended simply in order to hear comes at the cost of processing resources that might otherwise be available for encoding the speech content in memory [90, 91]. However, when NR was implemented, the negative effect of noise on recall was reduced, even though the ability to repeat sentence-final words remained the same [83]. This demonstrates that hearing aid signal processing can enhance memory processes underpinning speech communication. Informational masking was more disruptive of memory processing than energetic masking and was also more susceptible to the positive effect of NR [83]. However, it remains to be determined whether it is the semantic content or phonological structure of the informational masker that interacts with the ability of NR to improve memory for highly intelligible speech.

Speech communication under adverse conditions is likely to draw on cognitive functions other than simply memory retention [30, 59]. In order to investigate the ability to perform executive processing of heard speech at different memory loads, the cognitive spare capacity test (CSCT) [82, 84] was developed. In the CSCT, sets of spoken two-digit numbers are presented and the participant reports back certain numbers according to instructions. Two executive functions are targeted at two different memory loads. The executive functions in question are updating and inhibition, both of which are likely to be engaged during speech understanding in adverse conditions. Updating ability may be required to strategically replace the contents of WM with relevant material while inhibition ability may be brought into play to keep irrelevant information out of WM. Memory load depends on how many numbers need to be reported. In everyday communication, seeing the face of your communication partner can enhance speech perception by several dB [92]. Thus, in order to determine how visual cues influence CSC, the CSCT manipulates availability of visual cues. The CSCT can be administered in quiet or in noise and other manipulations introducing different kinds of signal processing are also possible.

Across three different studies including persons with and without hearing loss, an interesting pattern of results has emerged [69, 82, 84, 93]. Adults with normal hearing who perform the CSCT in quiet conditions have lower scores when they see the talker's face [82, 84]. This is probably because when target information is highly intelligible, visual cues provide superfluous information that causes distraction during performance of the executive tasks [82, 84]. Although this finding is contrary to the literature on speech perception, which demonstrates better performance in noise when the talker's face is visible, for individuals with normal hearing [94] and individuals with hearing impairment [95–97], it is in line with other lines of evidence showing that visual cues may increase listening effort [98, 99]. In particular, dual task performance is lower for audiovisual compared to auditory stimuli when intelligibility is equated across modalities [98, 99].

Adults with normal hearing who perform CSCT in noisy conditions do not show this pattern [82] and nor do older adults with raised hearing thresholds, even in quiet [93]. In these conditions, visual cues probably help segregate the target signal from internal or external noise, resulting in richer cognitive representations [82, 100]. Older adults with hearing loss demonstrate lower CSC than young adults, even with better SNR, adapted to provide high intelligibility [101] and individualised amplification, and this effect is most notable in noise and when memory load is high [69]. Although CSC and WM do not seem to be strongly related, there is evidence that age-related differences in WM and executive function do influence CSC [69, 93]. It remains to be seen how different kinds of hearing aid signal processing will interact with executive processing of speech with and without visual cues and whether training CSC can counteract age-related decline in its capacity or even improve CSC. Adaptive training based on CSCT processing may provide a means of improving the ability to understand speech under adverse conditions.

### 3.2. Phonological Representation

The ELU model describes the way in which the mapping of phonological structure of target speech onto phonological representations in the mental lexicon [102] is mediated by WM during speech understanding under adverse conditions [41]. We have seen that fast-acting WDRC distorts the speech signal in a way that may influence its phonological characteristics [22, 28–30]. In the short term, this may make it harder to match speech to representations, thus requiring more cognitive engagement to achieve speech understanding [41, 70, 73]. However, in the long term, when hearing aid users have had the opportunity to become accustomed to the way in which speech sounds different, phonological representations may alter to match incoming information. Some evidence of this has been found in cochlear implantees [103] and hearing aid users [30]. It is even possible that the new phonological representations based on processed speech may be more mutually distinct than the representations they replace based on less appropriate signal processing. The neural correlates of such changes in phonological representation due to habitual use of WRDC have yet to be investigated.

Lexical access is faster when phonological representations are easier to distinguish from each other [102, 104]. However, long-term severe acquired hearing impairment may lead to less distinct phonological representations [103]. This makes it harder to determine whether printed words rhyme with each other [105], especially when orthography is misleading [106]. For example, individuals with poor phonological representations due to severe long-term hearing impairment may be more unsure than their peers with normal hearing whether “pint” rhymes with “lint” or whether “blue” rhymes with “through.” However, good WM capacity can compensate for this deficit, albeit at the cost of long-term memory representations [106]. Compensatory processing by individuals with hearing impairment during visual rhyme judgment is associated with larger amplitude of the N2 component [107], indicating use of a compensatory strategy, possibly involving increased reliance on explicit mechanisms such as articulatory recoding and grapheme-to-phoneme conversion.

In summary, phonological structure of target speech material is not only influenced by speaker characteristics but also by distortion due to hearing aid signal processing. Phonological representations in the mental lexicon may be influenced by long-term effects of both hearing impairment and signal processing. Further, both of these may have distinct neural signatures. Measures designed to improve phonological distinctiveness of both target speech and phonological representations are likely to enhance CSC and support speech communication under adverse conditions. This deserves further investigation.

### 3.3. Semantic Context

Provision of semantic context can facilitate speech understanding under adverse conditions. This process engages language networks in left posterior inferior temporal cortex and inferior frontal gyri bilaterally [108]. Studies investigating the role of WM capacity in the benefit obtained from WDRC have indicated that the semantic content of the materials delivered for speech recognition may influence this relationship. For example, Rudner et al. [30] found that WM capacity was associated with speech understanding for individuals with hearing impairment using WDRC listening to matrix-type sentences [109, 110], but not Swedish HINT sentences [88, 89]. The Hagerman sentences are semantically coherent, but the five-word syntactic structure is always the same and each word comes from a closed set of ten appropriate items. Thus none of the items can be accurately predicted. The HINT sentences, by contrast, are diverse in length, syntactic structure and semantic coherence. It is likely that the constrained structure and content of the Hagerman sentences make guessing harder and thus increase reliance on the bottom-up information provided by the speech signal. However, it has been found that the benefit of having access to the temporal fine structure of the speech signal was greater for open set materials than for closed-set materials [111], indicating that the regular structure and closed set of matrix-like sentences can facilitate guessing. Future work should systematically investigate the interaction between the semantic coherence of the speech signal, hearing aid signal processing, and individual cognitive characteristics such as WM and CSC.

Text cues can facilitate speech understanding in noise when they match the semantic content of the auditory signal [112–116] and inhibit it when they are misleading [116]. Cue integration is supported by language networks including the inferior frontal gyrus and temporal regions [115]. Matching text cues also enhance the perceived clarity of degraded speech [63] and recently it was shown that this effect may be modulated by both lexical access speed and WM capacity [117]. WM capacity modulates the activation of networks involved in semantic processing [115] and also predicts the ability to inhibit misleading text cues during speech understanding in steady state noise [116] as well as the facilitation of speech understanding against a single talker background [112]. Recently, it has been shown that coherence and cues can have separate facilitatory effects on perceived clarity of degraded speech [117]. Future work should focus on determining the benefit of providing text cues for hearing aid users, for example, using automatic speech recognition [114] and how this interacts with the semantic coherence of the target speech, the availability of semantic content in the noise background, and individual cognitive skills. Imaging studies are likely to provide important information about the neurocognitive systems supporting these complex interactions. Aging and Communication

Sensory and cognitive functions decline with age [118, 119]. Sensory decline can be traced to physiological change, but the mechanisms behind cognitive change are more elusive, although both genetic and lifestyle factors have been implicated [118]. Several different theories attempt to explain the relation between sensory and cognitive decline. The common cause hypothesis [119] proposes that a general reduction in processing efficiency drives both phenomena. The information degradation hypothesis [120], on the other hand, claims than when sensory input is degraded, cognitive processing becomes less efficient as a result. Reserve theories suggest that the ability to cope with brain damage is related to premorbid brain size or cognitive ability [86]. The compensation-related utilization of neural circuits hypothesis [35] suggests that older adults compensate for less efficient processing by engaging more neural resources than younger adults when task load is still relatively low while brain maintenance theory [118] proposes that individual differences in the manifestation of age-related brain changes and pathology allow some people to show little or no age-related cognitive decline. All these theories are more or less sophisticated in their attempts to capture the relationship between physiological, sensory, and cognitive function in an aging perspective. The relations they describe suggest that keeping the brain healthy and providing it with better sensory input will facilitate speech understanding for individuals of advancing age. The theories that focus on a special role for cognition suggest that lowering cognitive load and enhancing CSC during speech communication may have special importance in later adulthood and even allow some older adults to function communicatively just as successfully as their younger counterparts.

Recent work has shown that older adults show less activation in auditory cortex than younger adults while listening to speech in noise, especially at poor signal to noise ratios and compensate by recruiting prefrontal and parietal areas associated with WM [36]. Epidemiological studies show that individuals with hearing loss are at increased risk of cognitive impairment and that rate of cognitive decline and risk of cognitive impairment are associated with severity of hearing loss [121]. Thus, hearing loss may result in decreasing CSC. No study has yet specifically addressed this issue. However, analysis of data from the Betula study of cognitive aging [122] demonstrated that hearing aid users with poorer hearing also had poorer long-term memory [123]. This applied even when the long-term memory task had no auditory component. However, degree of hearing loss was not associated with decline in WM. Importantly, there was no significant association between loss of vision and cognitive function. These results suggest that although hearing loss and cognitive decline are related, even in hearing aid users, the association may not apply across all cognitive domains. The challenge is to uncover the specific mechanisms behind age-related sensory and cognitive decline so that speech communication can be preserved into old age by optimizing cognitive capacity. This may involve a range of different interventions that target hearing through appropriate hearing aid fitting, enhance the role of other sensory modalities that can be exploited in communication, and capitalize on cognitive abilities by seeking to maintain and extend them.

## 4. Conclusion

Speech communication in adverse conditions makes specific demands on cognitive resources. In particular, WM capacity and executive function are engaged in unravelling the speech signal. This depletes CSC and leaving fewer resources for higher level processing of speech. CSC is influenced by cognitive load, noise, visual cues, and aging and can be enhanced by appropriate hearing aid signal processing. The phonological structure and semantic content of speech influence processing mechanisms and engagement of cognitive resources. Optimizing CSC is an important aim for preserving speech communication into old age. We have reviewed evidence suggesting that CSC may be enhanced by a number of means including cognitive training and providing the optimal balance between visual, phonological, and semantic information. Future research should focus on finding ways to optimize CSC.
